# Pentacam Indices in Photorefractive Keratectomy Surgery

**DOI:** 10.25122/jml-2020-0057

**Published:** 2020

**Authors:** Ghazal Maraghechi, Habib Ojaghi, Firouz Amani, Telma Zahirian Moghadam

**Affiliations:** 1. School of Medicine, Ardabil Azad University, Ardabil, Iran; 2. Department of Surgery, School of Medicine and Allied Medical Sciences, Imam Reza Hospital,Ardabil University of Medical Sciences, Ardabil, Iran; 3. School of Medicine and Allied Medical Sciences, Ardabil University of Medical Sciences, Ardabil, Iran; 4. Social Determinants of Health Research Center, Ardabil University of Medical Sciences, Ardabil, Iran

**Keywords:** Pentacam indices, PRK, refractive error

## Abstract

Refractive eye surgeries are one of the most non-emergent ophthalmic surgeries due to the effect on the reduction of refractive errors, increasing visual acuity, enhancing the quality of vision, and indirectly increasing the quality of life of patients. The aim of this study was to determine Pentacam indices in the patients who underwent photorefractive keratectomy (PRK) during 2014-2018, as well as to show their correlation with the type of refractive error. This descriptive cross-sectional study was performed on 2215 eyes of 1125 patients undergoing PRK surgery. The patients’ checklist, including demographic information, refractive index, keratometry, pachymetry, corneal surface zone indices, and progressive corneal thickness indices, was provided. All data were analyzed using the IBM SPSS software, version 25. The findings showed that there was a significant association between posterior corneal astigmatism (PCA) and anterior corneal astigmatism (ACA) (p=0.00). The mean Kmax front was recorded as 44.844 ± 1.58 D, which was significantly correlated with the type of refractive errors (p=0.00). According to the findings, there was a significant relationship between anterior chamber indices and refractive error types and their severity (p=0.00). There was also a significant correlation between the surface zone and keratoconus indices (i.e., index of surface variance - ISV, index of vertical asymmetry - IVA, index of height asymmetry - IHA, and minimum radius of curvature - Rmin) with refractive errors (p=0.00). The findings showed that some of the Pentacam indices could be related to the types of refractive errors in patients undergoing PRK surgery. Therefore, their evaluation is of great importance in this regard.

## Introduction

Regarding the prevalence of refractive errors, photorefractive keratectomy (PRK) has been the most common non-emergency ophthalmic surgery in the last two decades. These surgeries include the correction of myopia, hyperopia, and astigmatism and reduction of dependence on the use of glasses or contact lenses [1-4].

Long-term stability, efficacy, and predictability of PRK have been established in several studies [5]. However, ophthalmologists still face the challenge of identifying patients at risk for postoperative complications such as overcorrection, under-correction, ectasia, ad others [6, 7]. Thus, the use of devices such as Ultrasound, Galile, Orbscan, and Pentacam can minimize the errors caused by measurements of corneal indices. One of the most important and commonly used devices is Pentacam [8].

Accurate measurement of corneal parameters is critical in achieving an appropriate therapeutic strategy and preventing complications of PRK. Therefore, the determination of the topographic design for the anterior and posterior corneal curvature and pachymetry is the primary basis of diagnostic tests for refractive surgery. Also, a detailed database on the characteristics of the components of the visual system in each country is needed due to the influence of racial and geographic factors. This study aimed to evaluate Pentacam indices in patients undergoing PRK surgery at Noor Surgery Center in Ardabil for five years, from the beginning of 2014 until the end of 2018.

## Material and Methods

This descriptive cross-sectional study was performed on patients undergoing PRK surgery over 5 years (from 2014 to 2018) at Noor Surgery Center in Ardabil. Furthermore, the sampling was performed by the census method. Moreover, 1125 medical records and 2215 eyes were finally evaluated. Patients’ information such as demographic information (age, sex), refractive data, keratometric data, pachymetry data, and keratoconus (KCN) classification were extracted from patient records and included in a comprehensive checklist. All patients were preoperatively examined by a surgeon (Ojaghi H) and underwent surgery by the same surgeon. Refraction was performed using the Canon (Canon Full Auto Ref-keratometer RK-F2, Tokyo, Japan) 30 minutes after administering two drops of cyclopentolate, 5 minutes apart. Data were obtained from Pentacam eye examination (Oculus Instruments, Wetzlar, Germany), including pachymetry, tomography, and anterior chamber evaluation.

The following rules were used to determine the type of refractive, anterior and posterior corneal astigmatism according to the steep corneal axis range: with-the-rule (from 0º to 30º or from 150º to 180º , against-the-rule (from 60º to 120º) and oblique (from 31º to 59º and/or from 121º to 149º) [9, 10].

We used the following formula to determine the severity of corneal astigmatism [11]: corneal astigmatism= K2-K1.

Also, we divided the refractive errors by their severity.

Myopia was classified into four levels, as follows:

• Mild myopia: spherical equivalent (SE) <3 diopters (D)
• Moderate myopia: SE 3-6 D
• High myopia: SE 6.25 - 9 D
• Extreme myopia: SE > 9 D [12, 13].

Hyperopia was classified into three levels, as follows [14]:

• Mild hyperopia: SE <2 D
• Moderate hyperopia: SE 2.25 - 4.75 D
• High hyperopia: SE ≥5 D
• Astigmatism was classified as follows:
• Mild astigmatism: <1 D
• Moderate astigmatism: 1-2 D
• High astigmatism: 2.25 – 4 D
• Extreme astigmatism: > 4 D [13].

To reduce examiner’s mistakes and increase stability, refractive measurements were performed only by using an automatic refractometer.

Inclusion criteria included all patients referred to an ophthalmologist with clinical symptoms and examination consistent with refractive astigmatism, hyperopia, and myopia. Exclusion criteria included patients with a history of connective tissue disease, herpes keratitis, corneal scar, cataract, uveitis, uncontrolled diabetes, immunosuppressive diseases, and patients younger than 18 years.

### Ethical considerations

Written informed consent for prospective data analysis was obtained from the patients during the recruiting process. The study and consent procedure were approved by the Ethics committee of the Ardabil University of Medical Sciences (No: 11910101972034) and adhered to the principles of the declaration of Helsinki.

### Statistical analysis

Checklist information was entered into the IBM SPSS v25 software. Descriptive statistics were used to analyze the data in most tables, graphs, central tendency (mean, median, mode), and dispersion indices (variance, standard deviation, range). Statistical analysis was performed using statistical tests, including one-way ANOVA, t-test, and Pearson’s correlation coefficient. The pre- and post-PRK visual acuity did not enter the checklist because the aim of the study was the evaluation of Pentacam indices. A P-value <0.05 was considered statistically significant.

## Results

In this study, 2215 eyes of 1125 patients undergoing PRK surgery were studied. Of the 1125 patients, 378 (33.6%) were male, and 747 (66.4%) were female. The mean age of patients was 28.48 ± 6.82 years, with a mean age ranging from 18 to 52 years (mode: 24 years and median: 27 years). Of the studied eyes, 1111 (50.2%) were right eyes, and 1104 (49.8%) were left eyes.

The mean sphere of all samples was -3.39 ±2.55 D with a range of -10.5 to +8.5 D, and the mean refractive astigmatism was -1.03 ± 1.12 D (ranging between 0 and 6 diopters). The mean spherical equivalent among samples was -3.91 ±2.50 D (ranging between -10.75 and +7.50 diopters).

**Figure 1:**
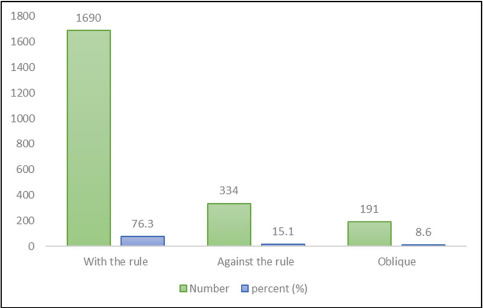
Frequency and percentage of refractive astigmatic types.

Figure 1 shows the percentage and frequency of refractive astigmatism based on the steepest meridian in the studied samples, in which the most common type of refractive astigmatism was with-the-rule (WTR) astigmatism (76.3%).

The frequency and percentage of anterior corneal astigmatism in 2215 eyes were: WTR astigmatism (82.7%; 1832 eyes) followed by against-the-rule (ATR) astigmatism (7.2%; 160 eyes) and oblique astigmatism (10.1%; 223 eyes) (Table 1).

**Table 1:** Corneal astigmatism types.

Parameters	With-the-rule	Against-the-rule	Oblique
Anterior cornea	1832 (82.7%)	160 (7.2%)	223 (10.1%)
Posterior cornea	2069 (93.4%)	44 (2%)	102 (4.6%)

Frequency and percentage of posterior corneal astigmatism (Table 1) were: WTR (93.4%; 2069 eyes), followed by ATR (2%; 44 eyes) and oblique (4.6%; 102 eyes).

Keratometric indices (anterior and posterior corneal surface) such as k1, k2, and k mean and anterior chamber indices are seen in Tables 2 and 3. Mean anterior corneal astigmatism was determined as -1.11 ± 1.14 D and mean posterior corneal astigmatism was -0.34 ± 0.18 D.

**Table 2:** Keratometric indices.

	Keratometric indices	Mean ± SD	Range
Anterior cornea	K^1^	43.09 ± 1.52	37.8 – 48.5
	K^2^	44.39 ± 1.52	39.7 – 50.1
	K^mean^	43.73 ± 1.45	39.2 – 49
	Astigmatism (K^2^- K^1^)	-1.116 ± 1.14	-6.1 – 4.9
	K^max^	44.84 ± 1.58	40.3 – 54.4
Posterior cornea	K^1^	-6.09 ± 0.24	(-7.2) – (-5.4)
	K^2^	-6.44 ± 0.27	(-7.5) – (-5.6)
	K^mean^	-6.26 ± 0.24	(-7.3) – (-5.6)
	Astigmatism (K^2^- K^1^)	-0.34 ± 0.18	-1.1 – 0.4

**Table 3:** Corneal pachymetry and anterior chamber indices.

Indices	Apex pachymetry (µm)	Thinnest location pachymetry (µm)	Cornea volume (mm^3^)	Chamber volume (mm^3^)	Anterior chamber depth (mm)	Anterior chamber angle (degree)
Mean ± SD	534.26 ± 32.04	531.27 ± 32.48	59.64 ± 3.61	201.62 ± 35.13	3.78 ± 0.30	37.94 ± 6.21
Range	434 - 687	429 - 680	49.5 – 76.2	94 - 336	2.67 – 6.68	15.1 – 79.5

Abbreviations: SD - standard deviation.

Pearson’s correlation test showed that there is a strong direct significant relationship between pachymetry of apex and the thinnest location (P = 0.00, r = 99%).

Of the total samples, 1899 (89.73%) exhibited myopia with a mean spherical equivalent (SE) of -4.07 ± 1.76, followed by astigmatism (234; 10.57%) and hyperopia (82; 3.7%) with a mean SE of +4.64± 1.67 D. Mean and standard deviation of SE of myopia, hyperopia and astigmatism were -4.45 ± 1.87, +4.01 ± 1.64 and -2.25 ± 1.60 D, respectively.

Myopia was found as mild in 27.96% cases, followed by moderate (56.5%), high (14.69%), and extreme (0.85%). Also, among the hyperopia patients, 6% showed low hyperopia, followed by moderate (47.6%) and high (46.4%) hyperopia. Among the patients with astigmatism, 0% had a mild type, followed by moderate (24.7%), high (54.7%), and extreme (20.8%).

The percentages of astigmatism types according to the location of the focal lines relative to the retina were as follows: simple: 60 (25.64%), compound: 148 (63.24%), and mixed: 26 (11.12%).

According to Table 4, the results showed significant associations of refractive errors of myopia, hyperopia, and astigmatism with mean thinnest location indices (p=0.048), Kmax front (p=0.00), cornea volume (p=0.006), anterior chamber (AC) depth, chamber volume, chamber angle, index of surface variance (ISV), index of vertical asymmetry (IVA), index of height asymmetry (IHA), and minimum radius of curvature (Rmin) (All: p=0.00). However, the significant relationship between refractive errors and thinnest location (p=0.048) was found to be weak. Furthermore, no significant relationship was found between refractive errors and indices of the pachy apex, index of height decentration (IHD,) pachymetric progression index maximum (Progmax), pachymetric progression index average (Progavg), pachymetric progression index minimum (Progmin), central keratoconus index (CKI), and keratoconus index (KI) (p>0.05).

**Table 4:** Comparison of Pentacam indices by refraction type.

	Refractive errors			
Pentacam indices	Myopia n=1899 (85.73%)	Hyperopia n=82 (3.70%)	Astigmatism n=234 (10.57%)	P-value
Thinnest location M ± SD	531.42 ± 31.87	536.23 ± 27.51	527.04 ± 34.22	0.048
Pachy apex M ± SD	534.39 ± 31.99	540.52 ± 27.69	530.94 ± 33.56	0.059
**K**_max_ **front** M ± SD	44.80 ± 1.54	44.19 ± 1.94	45.42 ± 1.63	0.00
Cornea volume M ± SD	59.72 ± 3.59	59.26 ± 3.21	59.11 ± 3.80	0.006
AC Depth M ± SD	3.80 ±0.28	3.38 ± 0.31	3.72 ± 0.39	0.00
Chamber volume M ± SD	204.52 ± 33.78	156.95 ± 35.07	191.75 ± 34.25	0.00
Chamber angle M ± SD	38.27 ± 6.13	33.62 ± 5.79	36.76 ± 6.36	0.00
ISV M ± SD	15.79 ± 4.95	19.91 ± 8.29	26.75 ± 7.82	0.00
IVA M ± SD	0.10 ± 0.06	0.14 ± 0.09	0.12 ± 0.05	0.00
IHA M ± SD	3.041 ± 2.386	3.850 ± 2.986	4.109 ± 3.327	0.00
IHD M ± SD	0.005 ± 0.009	0.007 ± 0.004	0.007 ± 0.003	0.08
**R**_min_ M ± SD	7.539 ± 0.286	7.665 ± 0.272	7.439 ± 0.268	0.00
**Prog**_min_ M ± SD	0.685 ± 0.128	0.672 ± 0.101	0.688 ± 0.130	0.62
**Prog**_avg_ M ± SD	0.953 ± 0.125	0.929 ± 0.117	0.960 ± 0.129	0.156
**Prog**_max_ M ± SD	1.180 ± 0.177	1.157 ± 0.150	1.191 ± 0.168	0.329
CKI M ± SD	1.007 ± 0.006	1.006 ± 0.005	1.007 ± 0.005	0.20
KI M ± SD	1.020 ± 0.031	1.015 ± 0.019	1.019 ± 0.018	0.247

Abbreviations: M - mean; SD - standard deviation; AC Depth - anterior chamber depth; ISV - index of surface variance; IVA - index of vertical asymmetry; IHA - index of height asymmetry; IHD - index of height decentration; R^min^ - minimum radius of curvature; CKI - central keratoconus index; KI - keratoconus index; Prog^min^ - pachymetric progression index minimum; Prog^avg^ - pachymetric progression index average; Prog^max^ - pachymetric progression index maximum.

## Discussion

The findings of this study showed that the mean age of the subjects in this study was 28.48 ± 6.82 years, with a minimum age of 18 and the maximum age of 52 years. Moreover, 66.4% of patients were female and 33.6% were male. Mean spherical equivalent among samples was found to be -3.91± 2.50 D.

Out of the 2215 eyes studied, myopia had the highest prevalence (85.73%), followed by astigmatism (10.57%) and hyperopia (3.70%).

Seyed Javad Hashemian **et al.** reported that 91.95% of the 2673 eyes of individuals screened for refractive surgery had myopia [15]. Another study by Heydari **et al.** indicated that out of the 400 eyes studied, myopia (94.2%) was the most frequent, whereas hyperopia (3.3%) was the least frequent. The average spherical equivalent was -3.29 ±2.27 D, which is consistent with the present study [16]. It is therefore expected due to the better response of myopia to PRK than other types of refractive errors. In the present study, the keratometric index for the posterior and the anterior corneal surface was calculated. According to the results, the mean of K1 for the anterior cornea was 43.097 ± 1.52 D, followed by the mean of the K1 for the posterior cornea (-6.099 ± 0.24 D), mean of the K2 for the anterior cornea (44.394 ± 1.52 D) and the mean of the K2 posterior cornea (-6.442 ± 0.27 D). Furthermore, the mean Kmax front was calculated as 44.844 ±1.58 D, where a significant relationship was found between this variable and the refractive errors (p=0.00) (Tables 2 and 4).

In this study, the mean Kmax front was determined to be 44.80 ± 1.54 D and 44.19 ± 1.94 D for myopia and hyperopia, respectively. In contrast, a similar study by Hashemi **et al.** aimed at assessing anterior chamber criteria in a study that included 283 eyes concerning the refractive status of patients. The team divided the patients into three groups - emmetropia, myopia, and hyperopia. Myopic eyes were then divided into four subgroups. The results of the mentioned study demonstrated that 85% of eyes had myopia, and no significant difference was observed between the Kmax Front and refractive errors (p = 0.1). Its mean was reported to be 45.03 ± 1.44 D for myopia and 44.3 ± 2.2 D for hyperopia [17]. However, the Kmax front in the myopia was also higher compared to hyperopia, but no significant difference was found, which can be justified by the high total power of the eyes, including the corneal power of the patients with myopia as compared to the subjects with hyperopia.

In the present study, the mean corneal thickness at the apex and thinnest locations were 531.279 ± 32.48 µm and 531.279± 32.48 µm, respectively. There was a significant and direct relationship between these two variables (P = 0.00). However, no significant relationship was found between these two variables with refractive errors (p=0.056 and p=0.048, respectively). In their study, Mohammadi **et al.** aimed to investigate refractive errors to find a significant relationship between the two variables of refractive error and corneal thickness in subjects with hyperopia [18]. A significant relationship was previously found between CCT (Central corneal thickness) and the severity of myopia [19]. The study by Mohammadi **et al.** reported a CCT of 533.22 ± 32.02 µm [18]. In a similar study by Hashemi **et al.**, no significant association between the thinnest location and corneal thickness with refractive errors was reported [17]. Another study demonstrated that the mean CCT was 549.5 ± 33.6 µm. However, no significant correlation was found between CCT and refractive errors [20].

In addition, fam **et al.** reported no correlation of CCT with the degree of myopia (21). Our finding is consistent with a number of other published studies, showing that CCT has no significant relationship with refractive errors (22-24). Therefore, the relationship of corneal thickness with refractive errors can be explained by racial or geographical differences in various populations.

In addition, the mean of anterior chamber indices was investigated in this study. The mean of anterior chamber angle, AC depth, anterior chamber volume, and cornea volume were 37.942 ± 6.2165°, 3.780 ± 0.3078 mm, 201.629 ± 35.1351 mm3 and 59.61 ±3.67 mm3, respectively.

The results indicated that anterior chamber indices were significantly correlated with refractive errors. Based on the results of this study, it was observed that the highest amount of data in AC depth, chamber volume, and AC angle was related to the myopia group.

The findings of another study by Razmjoo **et al.** in patients undergoing PRK surgery showed that the mean of anterior chamber angle, AC depth, and anterior chamber volume were 39.7 ± 9.2°, 3.29 ±0.4 mm and 207±50 mm3, respectively [25]. A study by Hashemi **et al.** found significant associations of anterior chamber depth, anterior chamber volume and angle with refractive errors. The highest values of data were observed in anterior chamber depth and volume variables in the myopia group. However, no significant relationship was found between cornea volume and refractive errors (p> 0.05) [17]. A study of 297 eyes of 149 patients using Pentacam revealed the highest prevalence of myopia (242 eyes). There was a significant association between all refractive errors with pachymetric and anterior chamber indices (p<0.05), where the lowest values of corneal volume and the highest depth and volume of anterior chamber belonged to the myopia group [26]. Another study showed a weak correlation between cornea volume and myopia severity [27]. Similar findings have also been reported by other studies [28-30]. Most of the above studies are consistent with the present study because of the larger size and volume of the eyes. Therefore, the anterior chamber depth and anterior chamber angle are higher in myopia than in hyperopia.

Other achievements of this study were the evaluation of surface zone indices and criteria for the diagnosis of keratoconus such as ISV, IVA, IHD, IHA, Rmin, CKI and KI, and their significant relationship with refractive errors. According to the results, the overall mean ISV index was determined (17.1079), followed by mean IVA (0.1075), mean IHA (3.1843), and Rmin (7.5339). The results showed a significant relationship between refractive errors and their severity levels with IVA, ISV, IHA and Rmin indices. However, no significant relationship of IHD, KI and CKI indices with refractive errors was found. Also, no data was found in the literature in this regard. Only in one study by Brizl and **et al.**, the mean ISV in 36 eyes of patients without keratoconus was 21.83 ± 8.03, followed by mean IVA (0.12 0 0.04), mean IHA (5.02 ± 4.29), and Rmin (7.18 ± 0.17) [31].

Progressive corneal thickness indices were also evaluated in this study, including Progmin, Progavg, and Progmax, with a mean of 0.685, 0.952, and 1. 180, respectively. There was no significant relationship between refractive errors and progressive corneal thickness indices. One study showed that there was no significant relationship between Progavg and refractive errors [17]. This can be justified by the normal pattern of gradual increase in corneal thickness from center to corneal periphery (limbus) in all refractive errors as well as in normal eyes.

Based on the findings of the present study, the mean anterior corneal astigmatism was -1.116 ± 1.14 D, and the mean posterior corneal astigmatism was -0.34 ± 0.18 D. The most common type of astigmatism in both the anterior and posterior cornea was with-the-rule. Furthermore, a significant relationship was found between refractive astigmatism and anterior corneal astigmatism. Anterior corneal astigmatism also showed a strong association with posterior corneal astigmatism. Posterior corneal astigmatism (PCA) values in the normal population range from 0.26 to 0.78 D [32-34]. Askari Zadeh **et al.** performed a retrospective case series consisted of 161 eyes of 161 patients with KCN at Farabi Hospital in Tehran. They reported that mean anterior corneal astigmatism (ACA) and PCA were 4.08 ± 2.21 D and 0.86 ± 0.45 D, respectively. Also, the prevalence of WTR in the anterior corneal surface and prevalence of ATR astigmatism in the posterior cornea were found to be significantly higher in this study.

In accordance with our findings, Nemeth **et al.** reported that the most common type of posterior and anterior astigmatism was WTR [35]. A significant relationship of PCA with ACA has been reported previously. Contrary to the findings of this study, the most common type of anterior corneal astigmatism was WTR, and the most frequent type of posterior astigmatism was ATR [36]. Additionally, a study indicated that WTR anterior and ATR posterior corneal astigmatisms were the most prevalent types [37]. In contrast to the present study, Miyake **et al.** observed no significant association between PCA and ACA. WTR in the anterior cornea (68%) and ATR astigmatism in the posterior cornea (91%) were found to be the most prevalent types [38]. The different results reported in various studies may also indicate differences in the type of anterior and posterior astigmatism in different races and geographical areas or unknown cases that require further studies.

## Conclusion

The findings of the present study in patients undergoing photorefractive keratectomy surgery showed that Pentacam indices (i.e., keratometric indices, anterior chamber indices, and surface zone indices) might depend on the types and severity of refractive errors. Therefore, a more accurate and effective evaluation of the anterior and posterior cornea is needed to obtain a more favorable outcome from refractive surgery.

## Conflict of Interest

The authors declare that there is no conflict of interest.
